# What predicts the proxy-reported health-related quality of life of adolescents with cerebral palsy in Bangladesh?

**DOI:** 10.1186/s12889-019-8130-1

**Published:** 2020-01-07

**Authors:** Rosalie Power, Claire Galea, Mohammad Muhit, Eamin Heanoy, Tasneem Karim, Nadia Badawi, Gulam Khandaker

**Affiliations:** 1 Discipline of Child and Adolescent Health, Sydney Medical School, the University of Sydney, Sydney, NSW Australia; 2 Asian Institute of Disability and Development (AIDD), University of South Asia, Dhaka, Bangladesh; 3 Cerebral Palsy Alliance Research Institute, University of Sydney, Sydney, NSW Australia; 4 CSF Global, Dhaka, Bangladesh; 5 Central Queensland Public Health Unit, Central Queensland Hospital and Health Service, Rockhampton, QLD Australia

**Keywords:** Cerebral palsy, Bangladesh, Health-related quality of life, Low-middle-income country (LMIC), Adolescent, Disability, Teenager, Determinants

## Abstract

**Background:**

The health-related quality of life (HRQoL) of adolescents with CP in low and middle-income countries is often poor, as is the case in Bangladesh. This exploratory study examined what factors predict the proxy-reported HRQoL of adolescents with CP in rural Bangladesh, a typical low- and middle-income country (LMIC).

**Methods:**

Adolescents with CP (10 to 18y) were identified using the Bangladesh Cerebral Palsy Register. HRQoL was assessed using the Cerebral Palsy Quality of Life-Teens proxy-report questionnaire (CPQoL-Teens), adolescent mental health using the Strengths and Difficulty Questionnaire (SDQ) and caregiver mental health using the Depression, Anxiety and Stress Scale (DASS-21). Theoretical and statistical interests (i.e. bivariate analysis, *p* < 0.05) identified potential predictors which were entered into hierarchical multiple linear regression (HMLR) models in order of clinical significance; HMLR related adolescent clinical characteristics, adolescent and caregiver mental health and proxies of socioeconomic status to CPQoL-Teens dimensions.

**Results:**

One hundred fifty-four adolescents with CP (mean age 15y 1mo, SD 1y 8mo, female 31.2%) participated in this study.

Twenty-four factors were identified to explore for relationship to adolescent proxy-reported HRQoL. Fifteen of the factors correlated to one or more CPQoL-Teens dimension; strongest correlation was between ‘feelings about functioning’ and motor impairment (*r* = 0.545). Nine were predictive of CPQoL-Teens dimensions; adolescent sex, school attendance, severity of motor impairment, hearing and speech impairment, mother’s education, primary caregiver depression and stress, and having a sanitary latrine at home resulting in score changes of between 0.79 (95% CI 0.24 to 1.35) to 35.1 (95% CI 6.03 to 64.22).

**Conclusions:**

Many of the factors predicting the proxy-reported HRQoL of adolescents with CP are amenable to intervention, and have the potential to improve adolescent wellbeing. Several determinants are priorities of the sustainable development goals (SDGs); these findings should inform resource prioritization to improve the wellbeing of adolescents with CP in Bangladesh and other LMICs.

## Background

Health-related quality of life (HRQoL), a subjective multidimensional concept for measuring the interaction between health status and physical, psychological, and social aspects of wellbeing, is an emerging focus in low and middle-income countries (LMICs) such as Bangladesh [1]. Particularly relevant to adolescents with cerebral palsy (CP), understanding of HRQoL can be used as an indicator of intervention outcomes and provide understanding of burden of disease [2]. Moreover, understanding of HRQoL in LMICs flips the switch on poverty, allowing for focus on what people have, rather than what they do not [3].

CP is the major cause of childhood physical disability worldwide although the majority of cases are in LMICs [4]. Estimated prevalence of CP in Bangladesh is 3.4 per 1000 children and is likely to be associated with more severe physical, cognitive and communication impairments [5]. Recent studies have determined that adolescents with CP in Bangladesh [6], and from other LMICs [7] perceive significantly lower HRQoL outcomes than peers without disability, according to both self- and proxy-reported measures, although little is known about determinants of HRQoL in these settings.

To date, the majority of HRQoL research has been conducted in high-income countries (HICs) and findings suggest it is predicted by a range of personal and environmental factors [8]. For example, a European study of 551 adolescents with CP found psychological problems and parenting stress predicted HRQoL whereas socio-demographic and impairment severity characteristics did not [9]. Other studies set in HICs have reported that impairment, in particular motor functioning, is predictive of physical wellbeing dimensions of HRQoL [10, 11]. These are important findings to inform HRQoL research in LMIC settings, however patterns of HRQoL appear to be different between HIC and LMIC [7, 8]. It is therefore reasonable to suggest that determinants of HRQoL will also differ between HIC and LMIC, requiring specific exploration.

Only a handful of studies from LMICs have examined determinants of HRQoL among children and adolescents with CP, of which only one has examined predictive relationships [7]. The Turkish study of 40 children with CP found that, among other factors, motor function was predictive of ‘physical wellbeing’ using the Paediatric Quality of Life Inventory [12]; other studies have confirmed an association between motor function and HRQoL [12–16]. Studies from LMICs have also indicated a relationship between HRQoL dimensions and factors such as child age [13], child sex [13], child education [17], family income [13], speech impairment [18], cognitive impairment [14, 18], visual impairment [14], epilepsy/ seizure disorder [14, 15, 18], caregiver age [16], and mother’s education [13]. However, due to the emerging nature of HRQoL research in LMICs many of these findings require further exploration and replication. Currently, to the best of our knowledge there is no research on determinants of HRQoL of adolescents with CP from Bangladesh.

The present study explores what factors predict the proxy-reported HRQoL of adolescents with CP in rural Bangladesh. We have explored factors either informed by previous research or considered relevant to the research context including priority areas of the sustainable development goals of which Bangladesh is an adoptee [19]. Moreover, mental health is a significant issue among adolescents with CP and caregivers in rural Bangladesh [6, 20] and are included as potential determinants in our analysis. We also explore proxies of socio-economic status related to housing infrastructure due to their potential impact on adolescent wellbeing in the study context.

## Methods

### Study design, setting and participants

This is an exploratory study part of the Bangladesh CP Health-related Quality of life (Bangladesh CPQoL) research project. Participants were considered eligible for this study if aged between 10 and 18 years old, a normative classification of adolescence in Bangladesh [21], and were registered with the Bangladesh Cerebral Palsy Register (BCPR) (*n* = 192 at the time of the present study).

BCPR is the first ongoing surveillance program of children with CP in an LMIC. The population-based register covers a defined geographical region of the Shahjadpur sub-district of Sirajganj in the northern part of Bangladesh and holds data on socio-demographic, clinical (including severity, aetiology, associated impairments and risk factors), nutrition, education and rehabilitation status of children and adolescents with CP in Bangladesh.

Key Informant Methodology described in Khandaker, Smithers-Sheedy [22] is used to identify children and adolescents for BCPR.

Adolescent HRQoL and mental health were proxy-reported by primary-caregivers (i.e. parent, grandparent, other relative or close adult friend who provided the majority of their care and support). Adolescents provided self-reported data as part of the broader Bangladesh CPQoL research project however this is not reported for the present study.

Informed verbal and written consent were obtained for all individual participants included in the study. In cases of illiteracy, written consent was obtained by thumbprint. For participants under 16 years, written consent was provided by a parent or guardian. This study adhered to STROBE guidelines and all methods described adhered to the ethical approvals provided by the Bangladesh Medical Research Council (BMRC/NREC/2013–2016/1165) and University of Sydney Human Research Ethics Committee (2016/646).

### Measures

#### Demographic and impairment characteristics: Bangladesh Cerebral Palsy Register (BCPR)

We extracted demographic and clinical information about adolescents from the BCPR database including age, sex, type of CP, severity of motor impairment using the gross motor function classification system (GMFCS), other associated impairments, school attendance and proxies of socio-economic status such as monthly family income, household crowding, access to running water and sanitation. Impairments were categorized as yes/ no based on existing diagnosis or presence of impairment during BCPR assessment. GMFCS is a five level classification system; children classified at GMFCS level 1 are independently ambulant whereas children at Level V require wheeled mobility [23]. BMI was calculated as weight divided by height and considered underweight if < 18.5. Type of housing was defined as Kutcha (houses made from mud, thatch or other organic materials, considered impermanent); semi-pucca housing (made with a combination of materials, considered semi-permanent); and pucca (made from brick, stone, timber or cement, considered permanent). Number of household members was divided by number of rooms to provide persons per room rate of crowding. Non-sanitary latrine was defined as a latrine that discharges into open space.

#### Health-related quality of life: Cerebral Palsy Quality of Life – Teens (CPQoL-Teens)

Adolescent HRQoL was assessed using the Bengali version of Cerebral Palsy Quality of Life-Teens proxy-report questionnaire (CPQoL-Teens) [24, 25]. CPQoL-Teens is a condition specific instrument that uses a nine point Likert scale to assess 88 items across seven dimensions; ‘general wellbeing and participation’, ‘communication and physical health’, ‘school wellbeing’, ‘social wellbeing’, ‘feelings about functioning’, ‘access to services’ (proxy-report only) and ‘family health’ (proxy-report only). Psychometric properties of the Bengali version CPQoL-Teens are available in Power, Akhter 2019 and outcome scores for this sample in Power, Muhit [6].

#### Adolescent mental health: Strengths and Difficulties Questionnaire

Adolescent mental health was assessed by proxy-reporters using the Bengali version of Strengths and Difficulties questionnaire (SDQ) [26]. SDQ is a brief behavioural screening tool that assesses ‘emotional symptoms’, ‘conduct problems’, ‘hyperactivity/ inattention’, ‘peer relationship problems’, and ‘pro-social behaviour’. Adolescent mental health scores for this sample are available in Power, Muhit [6].

#### Caregiver mental health: Depression, Anxiety and Stress Scale - 21

Caregiver mental health was assessed using the Bengali version of ‘Depression, Anxiety and Stress Scale’ (DASS-21) [27]. DASS-21 is a 21-item standardized self-report questionnaire designed to measure the negative related emotional states of ‘depression’, ‘anxiety’ and ‘stress’. Caregiver mental health scores for this sample are available in Power, Muhit [20].

### Statistical methods

Descriptive statistics were used to summarise the cohort. CPQoL-Teens scores were converted to values between 0 to 100 and mean dimension scores calculated by averaging the items in each dimension; SDQ scores were summed into dysfunction scales and ‘total difficulties score’ calculated by summing the scales; DASS-21 scores were summed into ‘depression’, ‘anxiety’ and ‘stress’ scales and multiplied by two according to the instrument protocol. Data was assessed for normality using Shapiro Wilk and visual inspection of residual plots.

Hierarchical multiple linear regressions (HMLR) were used to determine predictors of each CPQoL-Teens dimension. Potential predictor variables for each model were selected on the basis of theoretical and statistical interests; initially, the research team conducted a systematic review, to identify current knowledge about the self- and proxy-reported HRQoL of adolescents with CP in LMICs, see Power, King [7]. The team then discussed the clinical and contextual significance of potential factors and constructed a list of adolescent and caregiver characteristics and proxies of socio-economic status to explore for relationship to adolescent HRQoL. Bivariate analysis using Spearman’s correlation was then conducted to determine the relationship between each CPQoL-Teens dimension and the selected variables; correlations were considered small (≤0.49), medium (0.50 to 0.79), and large (≥0.80) [28]. Variables with statistically significant correlations (i.e. bivariate analysis, *p* < 0.05) were entered into each model order of clinical significance. Assumptions of linearity, homoscedasticity and normality were assessed through examination of Q-Q plots and Shapiro-Wilks; independence of observations assessed with Durbin-Watson statistic, and Multicollinearity assessed through correlation, tolerance and variance inflation factor (VIF) coefficients. No adjustment was made for multiple testing due to the investigative nature of the study. All statistical analysis was conducted using SPSS version 24 (IBM Armonk, NY, USA). A *p* value of < 0.05 was considered significant.

## Results

### Participant characteristics

One hundred ninety two adolescents with CP aged 10 to 18 years were enrolled in BCPR at the time of the present study. 154 (mean age 15y 1mo) agreed to participate of which 48 (31.2%) were female. Participation rate was 80.2%.

Characteristics of adolescents with CP and caregivers are provided in Table 1. Majority of adolescents had spastic type CP, of which quadriplegia and monoplegia/hemiplegia were most common; required wheeled mobility (i.e. GMFCS level III and above); and were underweight. More than half had cognitive impairment and more than two thirds had speech impairment. Majority had never received any rehabilitation or attended school, lived in kutcha housing (impermanent) and had limited infrastructure in the home such as lack of running tap water; approximately one third had either a non-sanitary latrine or no toilet facility.

**Table 1:** Participant socio-demographic and impairment characteristics

Adolescent characteristics	
Age	
Mean (SD)	15y 1mo (1y 8mo)
Sex (n,%)	
Female	48(31.2%)
Male	106(68.8%)
BMI	
Median (IQR)	17.0(4.7)
GMFCS (n,%)	
Level I	36(23.4%)
Level II	23(14.9%)
Level III	33(21.4%)
Level IV	20(13.0%)
Level V	41(26.6%)
Unknown	1(0.6%)
Type of CP (n,%)	
Spastic	
*Monoplegia/hemiplegia*	41(26.6%)
*Diplegia*	27(17.5%)
*Triplegia*	12(7.8%)
*Quadriplegia*	43(27.9%)
Hypotonia	3(1.9%)
Dyskinesia	14(9.1%)
Ataxia	1(0.6%)
Unknown/ unclassified	13(8.4%)
Associated impairment (n,%)	
Epilepsy	36(23.4%)
Cognitive impairment	87(56.5%)
Visual impairment	12(7.8%)
Hearing impairment	19(12.3%)
Speech impairment	103(66.9%)
Education	
None	115(74.7%)
Primary, secondary or above	39(25.3%)
Rehabilitation (any type) (n,%)	43(27.9%)
Caregiver characteristics	
Caregiver age	
Mean (SD)	39y 9mo(9y 9mo)
Caregiver sex (n,%)	
Female	126(81.8%)
Male	28(18.2%)
Caregiver education (n,%)	
None	86(55.8%)
Primary, secondary and above	68(44.2%)
Proxies of SES	
Monthly family income (BDT)	
BDT Median (IQR)	6000(5000)
USD Median (IQR)	60.94(59.12)
Housing permanence (n,%)	
Kutcha house (impermanent)	119(77.3%)
Semi-pucca house (semi-permanent)	20(13.0%)
Pucca house (permanent)	15(9.7%)
Household crowding	
Median people/ room (IQR)	3.0(1.7)
Source of drinking water (n,%)	
Tube well	149(96.8%)
Tap water	5(3.2%)
Sanitation (n,%)	
No toilet facility	3(1.9%)
Non-sanitary latrine	50(32.5%)
Sanitary latrine	101(65.6%)

### Relationships of HRQoL outcomes to characteristics

#### Bivariate analysis

Results of correlation analysis, see Table 2, provides important information about the variables that were considered for inclusion in the HMLR (e.g. adolescent and caregivers characteristics, socio-economic status) and their statistical relationship to the CPQoL-Teens dimensions (i.e. general wellbeing and participation, communication and physical health, school wellbeing, social wellbeing, feelings about functioning, access to services and family health). Significant correlations were observed for all CPQoL-Teens dimensions with small to moderate effect (*r* = 0.164 to 0.545), with exception of ‘school wellbeing’ which did not report any significant correlations (*p* > 0.05).

**Table 2:** Spearman’s rho correlation matrix of HRQoL to characteristics of adolescents with CP

	General wellbeing and participation	Communication and physical health	School wellbeing	Social wellbeing	Feelings about functioning	Access to services	Family health
Adolescent characteristics							
Age	−0.044	−0.016	− 0.244	− 0.115	0.012	0.123	− 0.027
Sex	0.138	0.152	0.081	0.005	0.055	.227**	0.050
BMI	−0.071	− 0.020	− 0.061	− 0.071	0.071	0.047	− 0.053
Motor impairment (GMFCS)	−.274**	−.199*	− 0.148	− 0.111	−.545**	− 0.046	− 0.132
Epilepsy	0.122	0.151	0.297	0.118	0.154	0.151	0.090
Cognitive impairment	.251**	.244**	0.232	.221**	.180*	0.084	.189*
Visual impairment	−0.012	−0.003	− 0.295	0.018	− 0.012	− 0.015	0.105
Hearing impairment	−0.028	0.002	0.195	0.054	−0.001	−0.020	.232**
Speech impairment	.280**	.302**	0.170	.211**	.330**	−0.030	0.151
Education	.379**	.328**	−0.028	.274**	.330**	−0.094	.245**
Service Access	0.044	0.015	−0.243	−0.011	0.102	−0.021	− 0.041
Mental health (SDQ)	−0.120	− 0.124	0.092	− 0.124	0.016	0.121	−.196*
Caregiver characteristics							
Mother age	−0.14	−0.089	0.162	−.168*	−0.035	0.038	−.172*
Father age	−0.152	−0.09	0.228	−0.082	− 0.072	−0.019	− 0.091
Mother education	.209**	0.158	0.093	.223**	0.076	0.063	.168*
Father education	0.113	0.052	0.04	0.144	−0.016	0.056	0.05
Depression	0.143	.168*	0.273	−0.045	0.148	.349**	−.218**
Anxiety	0.124	0.107	0.148	−0.049	0.087	.252**	−.272**
Stress	0.008	0.014	0.099	−0.108	0.008	.234**	−.356**
Proxies of SES							
Family income (monthly)	.165*	0.093	−0.096	0.123	0.032	0.062	.197*
Housing permanence	.173*	0.115	0.115	.185*	0.074	0.029	0.128
Household crowding	0.005	−0.019	0.006	0.013	−0.113	0.023	−0.021
Source of drinking water	0.038	0.068	0.181	0.144	−0.019	−0.058	0.026
Sanitation	.164*	0.069	−0.183	0.144	.207**	0.081	0.042

Variables: Sex: 0 = Female, 1 = Male; GMFCS: 1 = Level I, 2 = Level II, 3 = Level III, 4 = Level IV, 5 = Level V; Epilepsy, Intellectual disability, Hearing, Speech, Visual impairment: 0 = Yes, 1 = No; Education: 0 = None, 1 = Primary or above; Service access: 0 = Yes, 1 = None; Housing permanence 1 = Kutcha, 2 = Semi-pucca, 3 = Pucca; Source of drinking water 0 = Tube well, 1 = Tap water; Sanitation 1 = No toilet, 2 = Non-sanitary latrine, 3 = Sanitary latrine

* *p* < 0.050; ** *p* < 0.010

Twenty-four variables were tested; ‘General wellbeing and participation’ significantly correlated to 8 variables; school attendance, GMFCS, cognitive impairment, speech impairment, mother education, family income, housing permanence and sanitation; ‘Communication and physical health’ to 5; school attendance, GMFCS, cognitive impairment, speech impairment and caregiver depression; ‘Social wellbeing’ to 6; school attendance, cognitive impairment, speech impairment, mother age, mother education and housing permanence; ‘Feelings about functioning’ to 5; school attendance, GMFCS, cognitive impairment, speech impairment and sanitation; ‘Access to services’ to 4; being male, caregiver depression, anxiety and stress; and ‘Family health’ to 10; school attendance, GMFCS, hearing impairment, mental health, mother age, mother education, caregiver depression, anxiety and stress, family income.

#### Hierarchical multiple linear regression

HMLR was conducted to determine predictors of each CPQoL-Teens dimension. Assumptions of linearity, homoscedasticity and normality were met for all dimensions. Multicollinearity was present in three models however was met after anxiety and stress were removed from ‘access to services’, depression and anxiety from ‘family health’ and sanitation from ‘general wellbeing and participation’.

The final models for each dimension, see Table 3, explained 14.3 to 24.4% of variation in scores. Nine variables independently predicted the CPQoL-Teens dimensions; GMFCS Level 5, adolescent education and mother education predicted ‘general wellbeing and participation’. Adolescent education and caregiver depression predicted ‘communication and physical health’. Adolescent education, speech impairment and access to a toilet in the home were predictive of ‘feelings about functioning’. Adolescent sex and caregiver depression predicted ‘access to services’. Hearing impairment, adolescent education and caregiver stress predicted family health. No variables were found to be predictive of social wellbeing.

**Table 3:** Hierarchical multiple regression predicting CPQoL-Teens dimensions

Variable	Final Model: B (95% CI)	β
General wellbeing and participation		
GMFCS: LI	Reference	
GMFCS: LII	2.05 (−8.25 to 12.36)	0.03
GMFCS: LIII	−1.49 (− 10.92 to 7.95)	−0.03
GMFCS: LIV	−6.17 (− 17.45 to 5.12)	− 0.10
GMFCS: LV	− 10.36 (− 19.92 to − 0.80)	− 0.22*
Cognitive impairment	5.16 (−2.41 to 12.73)	0.12
Speech impairment	1.23 (−7.17 to 9.63)	0.03
Adolescent education: None	Reference	
Adolescent education: Primary or above	9.51 (0.52 to 18.49)	0.19*
Mother education: None		
Mother education: Primary or above	9.05 (2.16 to 15.93)	0.21*
Family income	0.00 (0.00 to 0.00)	−0.11
Housing permanence: Kutcha (impermanent)	Reference	
Housing permanence: Semi-pucca (semi-permanent)	5.68(−4.12 to 15.47)	0.09
Housing permanence: Pucca (permanent)	6.25 (−5.72 to 18.22)	0.08
R^2 =^ 0.244 F = 4.138**		
Communication and physical health		
GMFCS: LI	Reference	
GMFCS: LII	0.73 (−8.04 to 9.49)	0.01
GMFCS: LIII	3.35 (−4.69 to 11.39)	0.08
GMFCS: LIV	2.10 (−7.39 to 11.59)	0.04
GMFCS: LV	−3.58 (−11.70 to 4.54)	−0.09
Cognitive impairment	4.08 (−2.37 to 10.53)	0.11
Speech impairment	3.31 (−3.83 to 10.44)	0.09
Adolescent education: None	Reference	
Adolescent education: Primary or above	8.24 (0.74 to 15.74)	0.20*
Caregiver depression	0.79 (0.24 to 1.35)	0.22**
R^2 =^ 0.204 F = 3.486**		
Social wellbeing		
Cognitive impairment	5.77 (−1.28 to 12.82)	0.15
Speech impairment	2.48 (−5.45 to 10.41)	0.06
Education: None	Reference	
Education: Primary or above	5.89 (−2.29 to 14.08)	0.13
Mother age	−0.36 (− 0.87 to 0.16)	− 0.12
Mother education: None	Reference	
Mother education: Primary or above	5.25 (−1.37 to 11.87)	0.12
Housing permanence: Kutcha (impermanent)	Reference	
Housing permanence: Semi-pucca (semi-permanent)	4.45(−4.97 to 13.88)	0.08
Housing permanence: Pucca (permanent)	5.16 (−5.12 to 15.43)	0.08
R^2 =^ 0.143 F = 3.551**		
Feelings about Functioning		
Adolescent education: None	Reference	
Adolescent education: Primary and above	12.2 (1.65 to 22.84)	0.20*
Cognitive impairment	−1.5 (−10.89 to 7.94)	0.03
Speech impairment	14.6 (4.10 to 25.08)	0.25**
Sanitation: No toilet facility	Reference	
Sanitation: Non-sanitary latrine	25.3 (−4.34 to 54.93)	0.34
Sanitation: Sanitary latrine	35.1 (6.03 to 64.22)	0.61*
R^2 =^ 0.200 F = 7.381**		
Access to Services		
Sex: Female	Reference	
Sex: Male	10.63 (2.82 to 18.44)	0.20**
Caregiver depression	1.95 (1.22 to 2.68)	0.26**
R^2 =^ 0.199 F = 18.729**		
Family Health		
Cognitive impairment	3.44 (−4.14 to 11.02)	0.07
Hearing impairment	11.33 (0.57 to 22.09)	0.16*
Adolescent education: None	Reference	
Adolescent education: Primary or above	9.10 (0.53 to 17.66)	0.17*
Mental health	−0.30 (−1.01 to 0.42)	−0.07
Mother age	−0.44 (−1.02 to 0.13)	−0.12
Mother education: None	Reference	
Mother education: Primary or above	2.30 (−5.24 to 9.85)	0.05
Caregiver stress	−1.21 (−2.16 to − 0.25)	−0.21**
Family income	0.00 (0.00 to 0.00)	0.03
R^2 =^ 0.215 F = 4.954**		

* *p* < 0.050; ** *p* < 0.010

## Discussion

This study examined what factors predict the proxy-reported HRQoL of adolescents with CP in rural Bangladesh. We assessed adolescent impairment characteristics and mental health, caregiver mental health, and proxies of socio-economic status. Our findings identified fifteen factors correlated to HRQoL of which nine were predictive (e.g. impairment characteristics, adolescent and mother’s education, caregiver mental health, etc.). Majority of the identified factors are amenable to intervention and are priorities of the sustainable development goals such as ‘gender equality’, ‘quality education’ and ‘clean water and sanitation’.

Our study examined the relationship between proxy-reported HRQoL and adolescent sex. We found that sex was predictive of ‘access to services’ with a higher proportion of males reporting better outcomes in this dimension. This finding is unique, although caution is recommended in interpretation due to the high proportion of male participants in our sample. Other studies from LMICs have reported a non-significant relationship between HRQoL dimensions and participant sex [12, 15, 17, 29], with exception of a study of children with CP in India [14] which reported males to have poorer overall HRQoL. The apparent sex-bias in the present study may be understood by considering the broader socio-cultural context in Bangladesh. Prioritisation of males for health care and family resources is culturally prevalent, and is more marked for those living below the poverty line [30].

School attendance was a significant predictor of four CPQoL-Teens dimensions. Children with disability in Bangladesh have the right to attend school enshrined in legislation [31] however a range of issues often prevent participation, such as non-acceptance from the school, parent refusal and transport issues; moreover non-school attendance among adolescents with CP in rural Bangladesh is associated with severity of motor impairment, cognitive and/or speech impairment [6]. Over half of adolescents with CP in our study had mild to moderate CP (59.7% with GMFCS I-III) however only 25.3% of participants attended school. Our findings of poorer wellbeing among non-school attenders supports that of a Brazilian study into children with CP [17].

We examined the relationship between adolescent impairments and proxy-reported HRQoL. Adolescent motor impairment (GMFCS L5), hearing and speech impairment were predictive of one CPQoL-Teens dimension each. Previous studies from LMICs have indicated a relationship between motor function and physical wellbeing dimensions of HRQoL, although no relationship has been consistently reported regarding other associated impairments [7]. Whilst the impairments themselves may not typically be modifiable, address of social stigma in the case of hearing impairment [32], and provision of hearing aids or implementation of non-verbal communication methods [33] may assist to improve outcomes in these dimensions. Cognitive impairment was associated with reduced scores on numerous CPQoL-Teens dimensions, however this was not predictive.

Caregiver characteristics correlated to, or were predictive of CPQoL-Teens dimensions, notably mother’s age and education. Interestingly, the relationships of these factors were not significant for fathers. These findings have important implications for future intervention design; in Bangladesh mothers typically undertake the majority of caregiving and due to an absence of services and support they may hold the primary responsibility for their children’s rehabilitation/ physical therapy [34]. Investing in mother’s education and provision of targeted interventions through mothers has the potential to increase understanding of effective mechanisms for improving the HRQoL of adolescent with CP. Moreover, the present study confirmed previous reports that caregiver mental health is an important predictor of adolescent HRQoL [9, 35] although effect in the present study was small.

We examined the relationship of several proxies of socio-economic status and proxy-reported HRQoL. Notably family income, living in permanent housing and having a sanitary latrine correlated to, or were predictive of CPQoL-Teens dimensions. Personal hygiene is likely to be a challenge for adolescents with motor impairments in the absence of assistive devices. Over one third of adolescents lived in housing without a sanitary latrine, indicating a daily struggle, forcing dependence on caregivers for essential daily tasks, and potentially increasing vulnerability to neglect and abuse.

There are a number of strengths and limitations to the present study. To the best of our knowledge this is the first study using a population-based sample to examine predictors of proxy-reported HRQoL among adolescents with CP in an LMIC although the sample-size did not reach ascertainment and our sample was homogenous in terms of socio-economic status; possible limitations to the generalizability of our findings. Moreover, we examined numerous variables that are likely to have a unique effect on the proxy-reported HRQoL in LMICs although we examined potential predictors of proxy-reported HRQoL only. Due to the subjective nature of HRQoL proxies are at best able to guess the wellbeing of their adolescent and results should be treated with caution. Self-reported HRQoL data is ideal and has been reported elsewhere [6] however were not included in the present analysis. We recommend future replication of our study in other regions of Bangladesh may help to confirm our findings. Moreover, we recommend future research examine the effectiveness of interventions to improve adolescent HRQoL outcomes.

## Conclusions

The proxy-reported health-related quality of life of adolescents with CP in rural Bangladesh was predicted by nine factors, many of which are modifiable. Adolescent and maternal access to education, caregiver depression and stress, infrastructure in the home (sanitary latrines) were predictive of CPQoL-Teens dimensions and should be considered in intervention development and resource allocation due to their probable benefits to HRQoL. Moreover, consideration of socio-cultural factors in resource allocation such as sex-bias may be important to reduce gender-inequality. Specific interventions addressing wellbeing and participation of adolescents with severe motor impairment and address of stigma impacting adolescents with hearing and/or speech impairment may have the potential to improve adolescent wellbeing. Our findings should inform resource prioritization in order to improve the HRQoL of adolescents with CP in Bangladesh.

## Data Availability

The datasets used and/or analysed during the current study are available from the corresponding author on reasonable request.

## Abbreviations

BCPR: Bangladesh cerebral palsy register
BMI: Body mass index
CP: Cerebral palsy
CPQoL-Teens: Cerebral palsy quality of life – teenagers
GMFCS: Gross motor function classification system
HRQoL: Health-related quality of life
LMIC: Low- and middle-income country
SD: Standard deviation
